# Antibacterial and Antifungal Terpenes from the Medicinal Angiosperms of Asia and the Pacific: Haystacks and Gold Needles

**DOI:** 10.3390/molecules28093873

**Published:** 2023-05-04

**Authors:** Christophe Wiart, Geethanjali Kathirvalu, Chandramathi Samudi Raju, Veeranoot Nissapatorn, Mohammed Rahmatullah, Alok K. Paul, Mogana Rajagopal, Jaya Seelan Sathiya Seelan, Nor Azizun Rusdi, Scholastica Lanting, Mazdida Sulaiman

**Affiliations:** 1 Institute for Tropical Biology & Conservation, University Malaysia Sabah, Kota Kinabalu 88400, Malaysia; 2 Department of Medical Microbiology, Faculty of Medicine, University of Malaya, Kuala Lumpur 50603, Malaysia; 3 Research Excellence Centre for Innovation and Health Products (RECIHP), Walailak University, Nakhon Si Thammarat 80160, Thailand; 4 Department of Biotechnology & Genetic Engineering, University of Development Alternative, Dhaka 1207, Bangladesh; 5 School of Pharmacy and Pharmacology, University of Tasmania, Hobart, TAS 7001, Australia; 6 Faculty of Pharmaceutical Sciences, UCSI University, Kuala Lumpur 56000, Malaysia; 7 Department of Chemistry, Faculty of Science, University of Malaya, Kuala Lumpur 50603, Malaysia

**Keywords:** antibacterial terpenes, antifungal terpenes, medicinal plants, Asia, Pacific

## Abstract

This review identifies terpenes isolated from the medicinal Angiosperms of Asia and the Pacific with antibacterial and/or antifungal activities and analyses their distribution, molecular mass, solubility, and modes of action. All data in this review were compiled from Google Scholar, PubMed, Science Direct, Web of Science, ChemSpider, PubChem, and library searches from 1968 to 2022. About 300 antibacterial and/or antifungal terpenes were identified during this period. Terpenes with a MIC ≤ 2 µg/mL are mostly amphiphilic and active against Gram-positive bacteria, with a molecular mass ranging from about 150 to 550 g/mol, and a polar surface area around 20 Å². Carvacrol, celastrol, cuminol, dysoxyhainic acid I, *ent*-1β,14β-diacetoxy-7α-hydroxykaur-16-en-15-one, ergosterol-5,8-endoperoxide, geranylgeraniol, gossypol, 16*α*-hydroxy-cleroda-3,13 (14)Z-diene-15,16-olide, 7-hydroxycadalene, 17-hydroxyjolkinolide B, (20*R*)-3*β*-hydroxy-24,25,26,27-tetranor-5*α* cycloartan-23,21-olide, mansonone F, (+)-6,6′-methoxygossypol, polygodial, pristimerin, terpinen-4-ol, and α-terpineol are chemical frameworks that could be candidates for the further development of lead antibacterial or antifungal drugs.

## 1. Introduction

The phylogenic tree of Angiosperms comprises 11 major taxa or clades so far grouped into: (i) Basal Angiosperms (Protomagnoliids, Magnoliids, Monocots, and Eudicots), (ii) Core Angiosperms (Core Eudicots, Eudicots, Rosids, Fabids, and Malvids), and (iii) Upper Angiosperms (Asterids, Lamiids, and Campanuliids), [1]. Within each clade, secondary metabolites are synthesised as weapons against phytopathogenic bacteria and fungi. Phytoanticipins are present in plants before microbial challenge and phytoalexins are produced by plants under microbial attack [2]. Terpenes (from the Latin *terebinthina*: turpentine) are formed by the oligomerisation of isoprene units into four classes: monoterpenes (C_10_), sesquiterpenes (C_15_), diterpenes (C_20_), and triterpenes (C_30_), and within each class, different folding and cyclisation result in a wealth of chemical frameworks [3].

The outer structure of bacteria and fungi provides resistance to terpenes and other xenobiotics. Gram-negative bacteria, compared to Gram-positive bacteria, are more resistant to plant natural products and antibiotics because they are packed in a hydrophilic and negatively charged shield of lipopolysaccharides [4]. However, water, nutrients as well as hydrophilic, and, to a lesser extent, amphiphilic xenobiotics with a molecular mass below 600 g/mol, cross this outer layer through large transmembrane protein channels known as porins or aquaporins [5].

In most fungal species, the inner cell wall consists of a core of covalently attached branched β-(1,3) glucan and chitin [6]. The yeast cell wall is made of β-(1,3) glucan and mannoproteins and is negatively charged [7]. Yeasts are more sensitive to biocides because they have wall bud scars left after division [6]. Filamentous fungi are negatively charged, the wall is thicker, and much more complex, hence more resistant to antifungal natural products [6]. In addition, Gram-positive bacteria, Gram-negative bacteria as well as yeasts and filamentous fungi have pumps in their walls that efflux antimicrobials [8]. Five types of bacterial efflux pumps have so far been identified: ABC (ATP Binding Cassette), Resistance Nodulation cell-Division (RND), Major Facilitators (MF), Small Multidrug Resistance (SMR), and Multidrug and Toxic compound Extrusion (MATE). For instance, within MF is the multidrug transporter NorA, which effluxes fluoroquinolones out of the cytoplasm of *S. aureus* [9].

The golden era of antibiotics and antifungals is over and there is now the urgency to identify antimicrobial agents as well as efflux pump inhibitors with original chemical frameworks. Angiosperms, particularly those used to treat microbial infections in Asia and the Pacific, are a vast source of natural products with chemical structures completely different from conventional antimicrobials often coming from the prokaryotic world. These plants have been studied for about the last 60 years, resulting in the publication of a mammoth quantity of experimental data, but not a single antibiotic or antifungal for oral or parenteral use has come from Angiosperms.

This review therefore aims to answer the following questions: (i) What are the antibacterial and antifungal activities of each class of terpenes? (ii) What is the distribution of terpenes among the various clades of Angiosperms? (iii) What is the strength and spectrum of activity of terpenes? (iv) What is the influence of molecular mass? (v) What is the influence of solubility and polar surface area? (vi) What are the structure-activity relationships? (vii) What are the mechanism of action of terpenes? (viii) What are the antibiotic and/or antifungal potentiating effects of terpenes? All data in this review were compiled from Google Scholar, PubMed, Science Direct, Web of Science, ChemSpider, PubChem, and library searches from 1968 to 2022.

## 2. Monoterpenes

**Figure 1:**
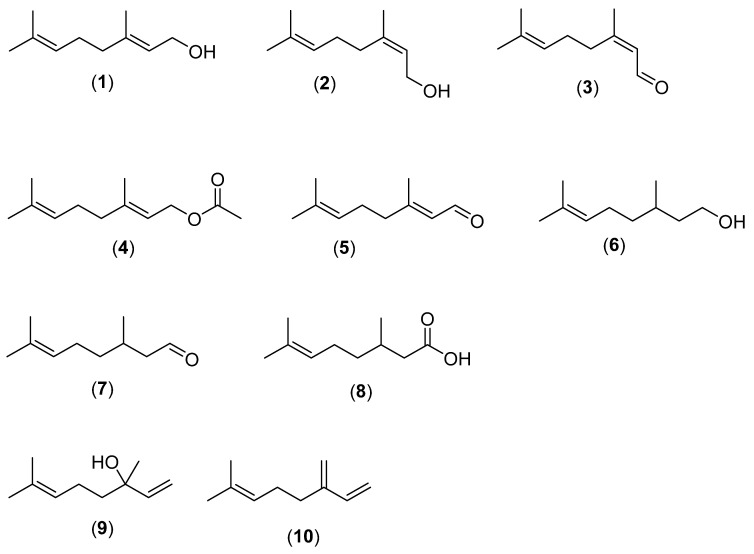
Antibacterial and antifungal linear monoterpenes. (**1**) geraniol, (**2**) nerol, (**3**) neral, (**4**) geranyl acetate, (**5**) geranial, (**6**) citronellol, (**7**) citronellal, (**8**) citronellic acid, (**9**) linalool, (**10**) myrcene.

Plants in the clades Magnoliids, Monocots, Malvids, and Lamiids produce volatile linear monoterpenes present in essential oils (Figure 1). Minimum inhibiting concentrations (MIC) are listed in Table S1.

### 2.1. Linear Monoterpenes

The condensation of dimethyl allyl diphosphate and isopentenyl diphosphate yields antibacterial and antifungal monoterpenes [10]. Examples are geraniol (**1**) (*Cymbopogon citratus* (DC.) Stapf.; Poaceae; Monocots), nerol (**2**), neral (**3**) (*Melissa officinalis* L.; Lamiaceae, Lamiids), geranyl acetate (**4**), geranial (**5**) (also known as citral), geranial (**6**), citronellol (**6**), citronellal (**7**) (*Eucalyptus citriodora* Hook.; Myrtaceae; Malvids), citronellic acid (**8**), linalool (*Cinnamomum bejolghota* (Buch.-Ham.) Sweet; Lauraceae; Magnoliids), and myrcene (**10**) (*Melaleuca alternifolia* Cheel; Myrtaceae) [11,12,13,14,15,16,17,18,19,20,21].

### 2.2. Cyclic Monoterpenes

**Figure 2:**
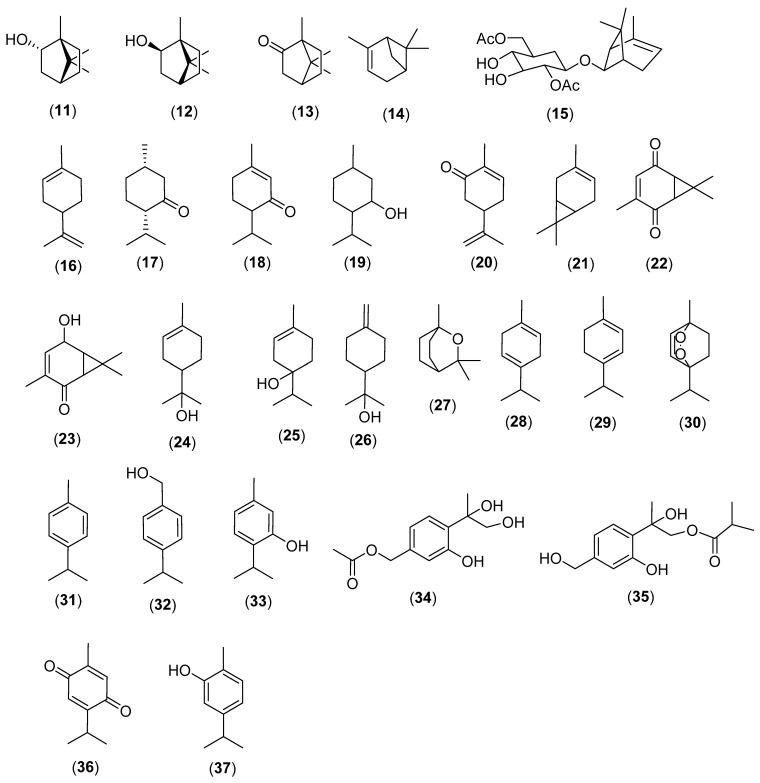
Antibacterial and antifungal cyclic monoterpenes. Borneol (**11**), isoborneol (**12**), camphor (**13**), α-pinene (**14**), α-pinene-7β-*O*-β-D-2,6-diacetylglucopyranoside (**15**), limonene (**16**), isomenthone (**17**), piperitone (**18**), menthol (**19**), carvone (**20**), car-3-ene (**21**), car-3-ene-2,5-dione (**22**), asarinol A (**23**), α-terpineol (**24**), terpinen-4-ol (**25**), δ-terpineol (**26**), 1,8-cineole (**27**), γ-terpinene (**28**), α-terpinene (**29**), ascaridole (**30**), *p*-cymene (**31**), cuminol (**32**), thymol (**33**), 7-acetyl-8,9-dihydroxy thymol (**34**), 7,8-dihydroxy-9-butyryl thymol (**35**), carvacrol (**37**).

Cyclic monoterpenes are antibacterial and antifungal such as borneol (**11**) (*Blumea balsamifera* (L.) DC.; Asteraceae; Campanuliids), isoborneol (**12**) (*Curcuma wenyujin* Y.H. Chen & C. Ling; Zingiberaceae; Monocots), camphor (**13**) (*Dryobalanops aromatica* C.F. Gaertn.; Dipterocarpaceae; Malvids), α-pinene (**14**) (*Altingia excelsa* Noronha; Altingiaceae; Core Eudicots), α-pinene-7β-*O*-β-D-2,6-diacetylglucopyranoside (**15**) (*Blumea lacera* (Burm. f.) DC.), limonene (**16**), isomenthone (**17**) (*Mentha piperita* L.; Lamiaceae), piperitone (**18**), menthol (**19**), carvone (**20**) (*Mentha spicata* L.), car-3-ene (**21**), car-3-ene-2,5-dione (**22**), and asarinol A (**23**) (Figure 2) [11,22,23,24,25,26,27,28,29,30,31].

α-Terpineol (**24**) in *Thymus vulgaris* L. (Lamiaceae) is a broad-spectrum antibacterial [20,32] as well as terpinen-4-ol (**25**) and δ-terpineol (**26**) (*Cinnamomum longepaniculatum* (Gamble) N. Chao ex H.W. Lide), 1,8-cineole (**27**) (*Eucalyptus globulus* Labill.) [13,17,18,32], γ-terpinene (**28**), α-terpinene (**29**), *p*-cymene (**31**), and cuminol (**32**) (*Cuminum cyminum* L.; Apiaceae) [32,33,34,35,36,37,38].

Thymol (**33**) in *Trachyspermum ammi* (L.) Sprague (Apiaceae; Campanuliids) is active against a broad-spectrum of bacteria and fungi [29,36]. 7-Acetyl-8,9-dihydroxy thymol (**34**) and 7,8-dihydroxy-9-butyryl thymol (**35**) from *Lonicera japonica* Thunb. (Caprifoliaceae; Campanuliids) are antibacterial [39]. Thymoquinone (**36**) (*Nigella sativa* L.; Ranunculaceae; Eudicots) [40,41,42,43] and carvacrol (**37**) (*Origanum vulgare* L.; Lamiaceae) are broad-spectrum antibacterial and antifungals [29].

## 3. Sesquiterpenes

MIC are listed in Table S1.

### 3.1. Linear Sesquiterpenes

**Figure 3:**
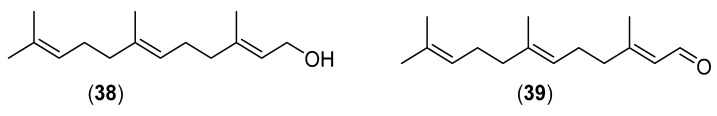
Antibacterial and antifungal linear sesquiterpenes. Farnesol (**38**), farnesal (**39**).

Farnesol (**38**) and farnesal (**39**) are antibacterial and antifungal and originate from the condensation of geranyl diphosphate and isopentenyl pyrophosphate [14] (Figure 3).

### 3.2. Cyclic Sesquiterpenes

**Figure 4:**
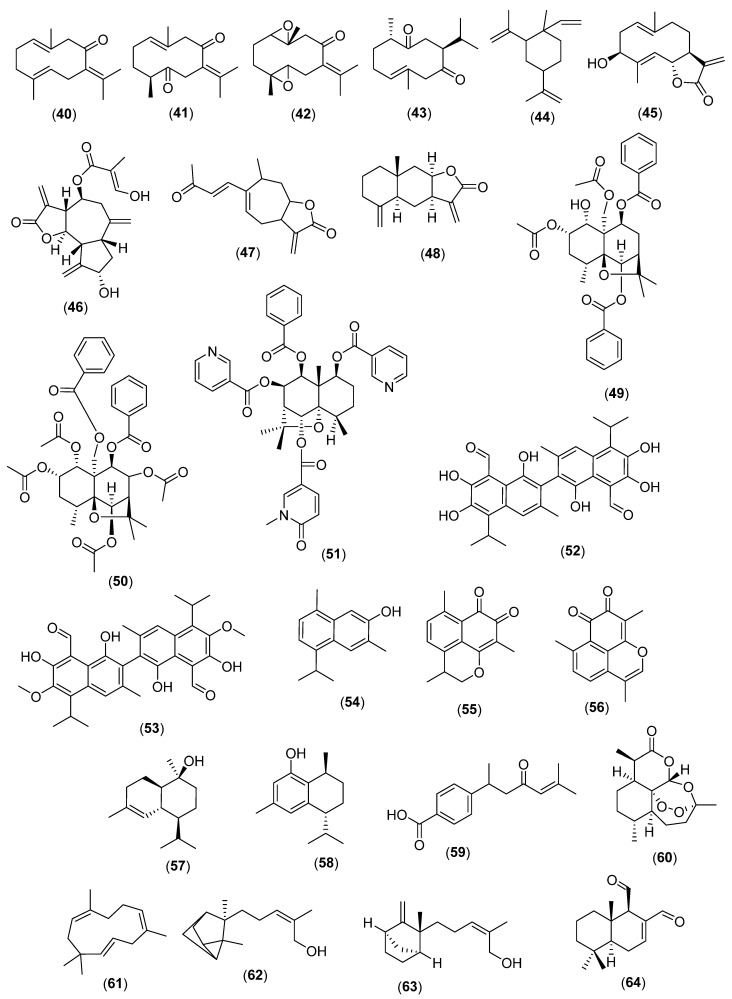
Antibacterial and antifungal cyclic sesquiterpenes. Germacrone (**40**), dehydrocurdione (**41**), 1(10),4(5)-diepoxygermacrone (**42**), c urdione (**43**), β-elemene (**44**), costunolide (**45**),cynaropicrin (**46**), deacetylxanthumine (**47**), isoalantolactone (**48**), microjaponin (**49**), 8-acetoxymutangin (**50**), monichinine H (**51**), gossypol (**52**), (+)-6,6′-methoxygossypol (**53**), 7-hydroxycadalene (**54**), mansonone E (**55**), mansonone F (**56**), cedrelanol (**57**),(+)-8-hydroxy calamenen (**58**), 4-(1,5-dimethyl-3-oxo-4-hexenyl) benzoic acid (**59**), artemisinin (**60**), α-humulene (**61**), α-santalol (**62**), β-santalol (**63**), and polygodial (**64**).

Cyclic sesquiterpenes originate from the farnesyl and nerolidyl cations [44] (Figure 4).

Plants in the Zingiberaceae and Costaceae families (Monocots) produce germacrane sesquiterpenes [44,45], which are on average broad-spectrum antibacterials such as germacrone (**40**), dehydrocurdione (**41**), and 1(10),4(5)-diepoxygermacrone (**42**) (*Curcuma heyneana* Valeton & Zijp) [46]. Germacrone (**40**), curdione (**43**), and β-elemene (**44**) in *C. wenyujin* are active against *Malassezia furfur* (ATCC 44344) [37]. Costunolide (**45**) (*Costus speciosus* (J. Koenig ex Retz.) Sm.) is antifungal [47]. Other antibacterial germacrane sesquiterpenes are found in the Burseraceae (Malvids) and Asteraceae [48,49].

Plants in the Magnoliids, Monocots, Malvids, Lamiids, and Campanuliids produce antibacterial and antifungal guaiane sesquiterpenes [50]. These are found, for instance, in *C. speciosus* [47] or *Syzygium cumini* (L.) Skeels (Myrtaceae) [24], and *Cynara scolymus* L. (also known as artichoke) (Asteraceae), the latter producing the anticandidal cynaropicrin (**46**) [51]. Other guaiane sesquiterpenes have been identified from *Torilis japonica* (Houtt.) DC. *Ferula diversivittata* Regel & Schmalh., both in the Apiaceae [52,53]. An example of the antifungal xanthanes is deacetylxanthumine (**47**) (*Xanthium strumarium* L.; Asteraceae) [54].

Broad-spectrum antibacterial and antifungal eudesmane sesquiterpenes found, for instance, in *Laurus nobilis* L. *Cinnamomum cassia* (L.) J. Presl (Lauraceae) [55,56,57]. Isoalantolactone (**48**) in *Abutilon indicum* (L.) Sweet (Malvaceae; Malvids) and *Inula racemosa* Hook f. (Asteraceae) inhibited *Aspergillus flavus*, *Aspergillus niger*, *Geotrichum candidum*, *Candida tropicalis*, *Candida albicans*, *Gaeumannomyces graminis, Rhizoctonia cerealis*, *Phytophthora capsici*, *Bacillus subtilis, Escherichia coli*, *Pseudomonas fluorecens, Sarcina lutea*, and *Staphylococcus aureus* [58]. In the Malvids, *Aquilaria sinensis* (Lour.) Spreng (Thymeleaceae) has antibacterial and antifungal eudesmanes [59].

Plants in the Celastraceae produce a unique series of antimycobacterial and antifungal dihydroagarofuran sesquiterpenes such as microjaponin (**49**) (*Microtropis japonica* (Franch. & Sav.) Hallier f.) [60,61], 8-acetoxymutangin (**50**) [60], and monichinine H (**51**) (*Monimopetalum chinense* Rehder) [62,63,64].

Antibacterial and antifungal cadinane sesquiterpenes are common in the Bombacaceae, Malvaceae, and Sterculiaceae (Malvids) [65,66]. In *Gossypium arboreum* L. (Malvaceae), gossypol (**52**) is specifically active against Gram-positive bacteria and fungi [67,68]. 7-Hydroxycadalene (**54**), gossypol (**52**), and (+)-6,6′-methoxygossypol (**53**) inhibited the growth of Gram-positive bacteria including Vancomycin-resistant *Enterococcus faecium* [62,63,64,65,66,67,68,69,70].

Mansonone E (**55**) is fungicidal and mansonone F (**56**) (*Helicteres angustifolia* L. (Sterculiaceae; Malvids) is active against MRSA [71]. Other examples are cedrelanol (**57**) (*Commiphora* Jacq) [67,72] or (+) 8-hydroxy calamenene (**58**) in the genus *Dysoxylum* Bl. (Meliaceae) [73,74]. Cadinane sesquiterpenes in *Polygonum viscosum* Buch.-Ham. ex D. Don (Polygonaceae; Malvids) are active against resistant strains of *E. coli* and MRSA [75,76].

An example of antifungal bisabolane sesquiterpene is 4-(1,5-dimethyl-3-oxo-4-hexenyl) benzoic acid (**59**) (*Bridelia retusa* (L.) A. Juss.; Phyllanthaceae; Fabids), which is active against *Cladosporium cladosporioides* [76]. *Rudbeckia laciniata* L. (Asteraceae) produces antimycobaterial bisabolane endoperoxide sesquiterpenes [77]. Another instance is artemisinin (**60**) (*Artemisia annua* L.; Asteraceae) with *V. cholerae* [78].

α-Humulene (**61**) (*Premna integrifolia* L.; Verbenaceae; Lamiids) is a broad-spectrum antibacterial [79,80]. Bactericidal and anticandidal humulanes occur in *Zingiber cassumunar* Roxb. (Zingiberaceae) and *Psidium guajava* L. (Myrtaceae) [81,82,83,84,85].

### 3.3. Miscellaneous

These are lindenane sesquiterpene (*Chloranthus japonicus* Siebold; Chloranthaceae; Protomagnoliids) [86], α-santalol (**62**) and β-santalol (**63**) (*Santalum album* L.; Santalaceae; Malvids) [87,88,89], *allo*-aromadendranes (*Chisocheton penduliflorus* Planch. ex Hiern; Meliaceae), polygodial (bactericidal) (**64**) (*Polygonum hydropiper* L.; Polygonaceae) [90,91,92], cyclofarneanes (*Premna* L. (Verbenaceae) [93], and unusual sesquiterpenes in *Glyptopetalum calocarpum* (Kurz) Prain (Celastraceae) that are active against Gram-positive bacteria and *Microsporum gypseum* [94].

## 4. Diterpenes

MIC are listed in Table S1.

### 4.1. Linear Diterpenes

**Figure 5:**
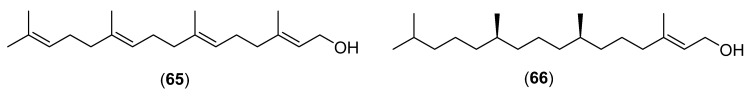
Antibacterial and antifungal linear diterpenes. Geranylgeraniol (**65**), (*E*)-phytol (**66**).

The addition of an isoprene to farnesol diphosphate yields geranylgeranyl disphosphate from which geranylgeraniol (**65**) is derived, active against *S. aureus* (FDA209P) [95] (Figure 5). Partial reduction in geranylgeranyl disphosphate forms (*E*)-phytol (**66**) (*Morinda citrifolia* L.; Rubiaceae; Lamiids) inhibited the growth of *S. aureus* (FDA209P) [87] and *Mycobacterium tuberculosis* (H37Rv) (MIC: 32 µg/mL) [96].

### 4.2. Cyclic Diterpenes

**Figure 6:**
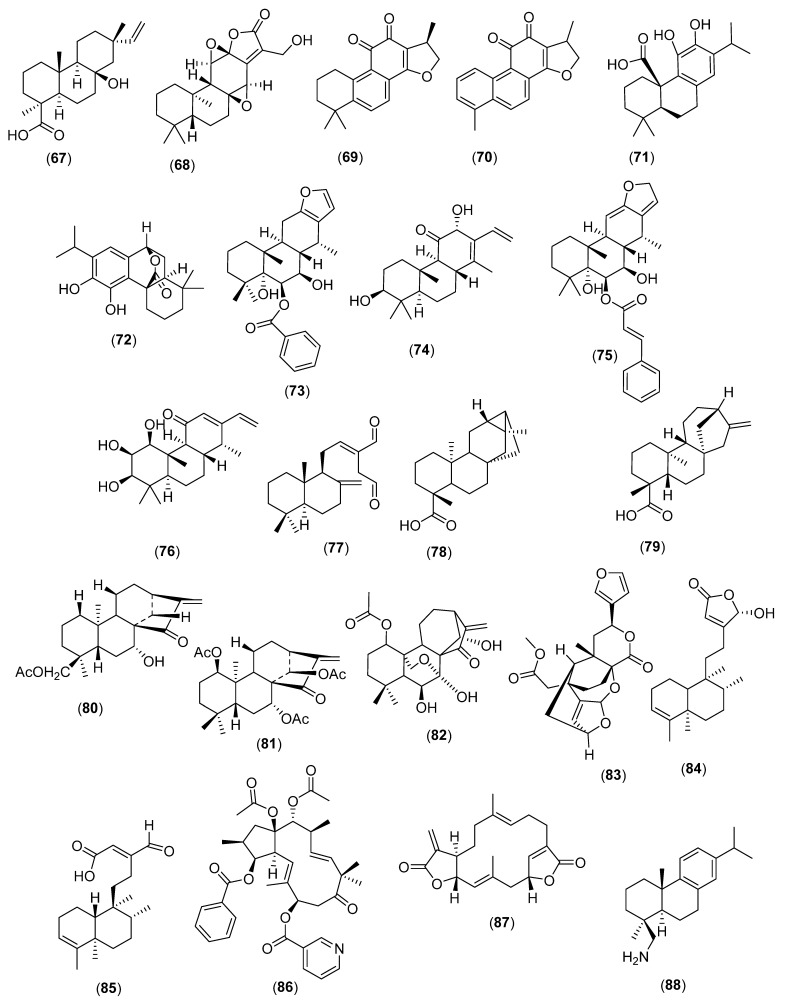
Antibacterial and antifungal cyclic diterpenes. Toonaciliatin M (**67**), 17-hydroxyjolkinolide B (**68**), cryptotanshinone (**69**), dihydrotanshinone I (**70**), carnosic acid (**71**), carnosol (**72**), is 6*β*-cinnamoyl-7β-hydroxyvouacapen-5*α*-ol (**73**), Niloticane (**74**), neocaesalpin P (**75**), phytocassane B (**76**), *E*)-8β, 17-epoxylabd-12-ene-15,16-dial (**77**), *ent*-trachyloban-19-oic acid (**78**), *ent*-kaur-16-en-19-oic acid (**79**), *ent*-18-acetoxy-7α-hydroxykaur-16-en-15-one (**80**), *ent*-1β,14β-diacetoxy-7α-hydroxykaur-16-en-15-one (**81**), lasiodin (**82**), bafoudiosbulbin C (**83**), 16*α*-hydroxy-cleroda-3,13 (14)Z-diene-15,16-olide (**84**), 16-oxo-cleroda-3, 13(14) *E*-diene-15 oic acid (**85**), euphoheliosnoid E (**86**), ovatodiolide (**87**), and dehydroabietylamine (**88**).

The cyclization of geranylgeranyl diphosphate accounts for the formation of all antibacterial and antifungal cyclic diterpenes (Figure 6).

Pimarane diterpenes in *Ceriops tagal* (Perr.) C.B. Rob. (Rhizophoraceae; Fabids) or *Toona ciliata* M. Roem. (Meliaceae) such as toonaciliatin M (**67**) are active towards *Trichophyton rubrum* (MIC: 12.5 µg/mL). Abietane diterpenes such as 17-hydroxyjolkinolide B (**68**) in *Euphorbia fischeriana* Steud. (Euphorbiaceae; Fabids) are active against *Mycobacterium smegmatis* [89,97,98,99]. Cryptotanshinone (**69**) and dihydrotanshinone I (**70**) from *Salvia miltiorrhiza* Bunge (Lamiaceae) are antibacterial and antifungal [100]. Of note, dihydrotanshinone I (**70**) protected mice against *C. albicans* at a dose of 5 mg/kg [101]. Carnosic acid (**71**) and carnosol (**72**) from *Rosmarinus officinalis* L. (Lamiaceae) are antibacterial against oral pathogens and are anticandidal [102,103,104].

Plants in the Fabaceae bring to being antimycobacterial furanoditerperne cassanes such as 6*β*-cinnamoyl-7β-hydroxyvouacapen-5*α*-ol (**73**) (*Caesalpinia pulcherrima* (L.) Sw.) [105]. Niloticane (**74**), in *Acacia arabica* (Lam.) Willd. [106] and neocaesalpin P (**75**) (*Caesalpinia bonduc* (L) Roxb.), is broadly antibacterial [107]. *Oryza sativa* L. (Poaceae), when experimentally challenged with *Magnaporthe grisea*, generates phytocassane B (**76**), active against the germination of and prevention of the spore germination of *M. grisea* (ED_50_: 4 μg/mL) [108]. Labdane diterpenes are broad-spectrum antibacterial and antifungal principles such as (*E*)-8β, 17-epoxylabd-12-ene-15,16-dial (**77**) in *Alpinia nigra* (Gaertn.) B.L. Burtt (Zingiberaceae) [109,110]. Anti-staphylococcal scopadulanes [96] are found in *Scoparia dulcis* L. (Scrophulariaceae; Lamiids) [111,112,113].

*Mitrephora celebica* Scheff. (Annonaceae; Magnoliids) produces *ent*-trachyloban-19-oic acid (**78**), active against oral pathogens *Streptococcus mutans* and *Porphyromonas gingivalis* bacteria as well as antimycobacterial kauranes such as *ent*-kaur-16-en-19-oic acid (**79**) [114,115,116,117,118]. The growth of *M. tuberculosis* (H37Ra) was inhibited by *ent*-18-acetoxy-7α-hydroxykaur-16-en-15-one (**80**) and *ent*-1β,14β-diacetoxy-7α-hydroxykaur-16-en-15-one (**81**) (*Croton tonkinensis* Gagnep. (Euphorbiaceae) [119]. Another instance is lasiodin (**82**) in *Rabdosia serra* (Maxim.) H. Hara (Lamiaceae) [120].

Clerodanes are, in general, antibacterials such as bafoudiosbulbin C (**83**) (*Dioscorea bulbifera* L.; Dioscoreaceae), active against *Salmonella s* [121,122,123]. Other examples are 16*α*-hydroxy-cleroda-3,13 (14)Z-diene-15,16-olide (**84**) and 16-oxo-cleroda-3, 13(14) *E*-diene-15 oic acid (**85**) (*Polyalthia longifolia* (Sonn.) Thwaites; Annonaceae), the latter being active against *Sporothrix schenckii* [124,125].

Plants in the Lecythidaceae, Verbenaceae, and Euphorbiaceae such as *Croton laui* Merr. & F.P. Metcalf produce antibacterial clerodane diterpenes [126,127,128,129]. Euphorbiaceae produce antifungal and antimycobacterial jatrophanes and tiglianes [130,131,132] such as euphoheliosnoid E (**86**) (active against Gram-positive bacteria *S. mutans* (ATCC 25175) and *Actinomycetes viscosus* (ATCC 27044) [133,134].

Plants in the Lamiaceae yield antimycobacterial cembranes, one example being ovatodiolide (**87**) from *Anisomeles indica* (L.) O.K.(IC_90_: 6.5 μg/mL) [135].

### 4.3. Miscellaneous

These are mainly found in Basal Angiosperms (Alismataceae) such as ent-rosanes, with diterpenes active against oral pathogens [118], antibacterial linear diterpene glycosides in Crocus sativus L. (Iridaceae; Monocots) [136,137], and antifungal alkaloid diterpenes from Delphinium denudatum Wall. ex Hook. f. & Thomson (Ranunculaceae; Eudicots) [138]. Aster triplolium L. (Asteraceae) produces broad-spectrum antibacterial abietane diterpene alkaloids such as dehydroabietylamine (**88**) [139].

## 5. Triterpenes

MIC are listed in Table S1.

**Figure 7:**
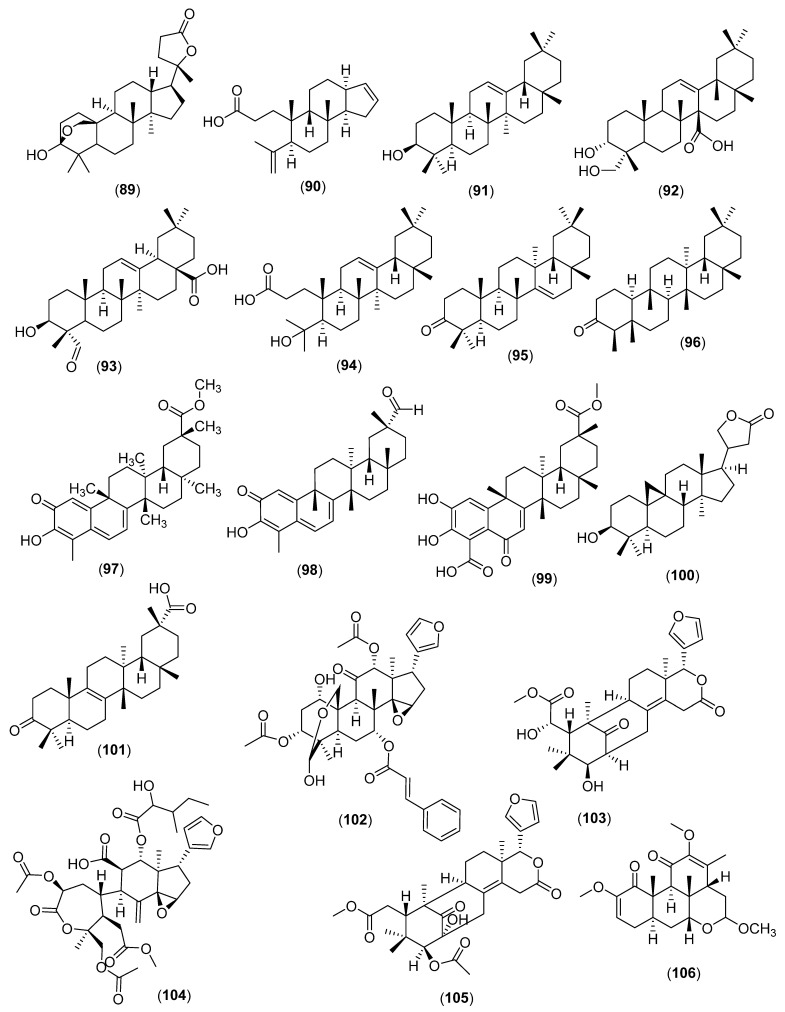
Antibacterial and antifungal cyclic triterpenes. 4-*Seco*-mansumbinoic acid (**90**), β-amyrin (**91**), aceriphyllic acid A (**92**), gypsogenin (**93**), dysoxyhainic acid I (**94**), taraxerone (**95**), friedelin (**96**), pristimerin (**97**), celastrol (**98**), zeylasterone (**99**), (20*R*)-3*β*-hydroxy-24,25,26,27-tetranor-5*α* cycloartan-23,21-olide (**100**), bryononic acid (**101**), 7-cinnamoyltoosendanin (**102**), swietenolide (**103**), mulavanin D (**104**), 2-hydroxyfissinolide (**105**), (6*R*)-methoxyjavanicin B (**106**), ergosterol-5,8-endoperoxide (**107**), stigmasterol 3–*O*–β–*D*–glucopyranoside (**108**), and dioscin (**109**).

The condensation of a pair of farnesyl cations forms 2.3-oxidosqualene, from which all antibacterial and antifungal triterpenes are derived by cyclisation (Figure 7).

### 5.1. Cyclic Triterpenes

Dammarane triterpenes such as amblyone (**89**) (*Amorphophallus paeoniifolius* (Dennst.) Nicolson; Araceae; Monocots) and 3,4-*seco*-mansumbinoic acid (**90**) (*C. wightii*) are antibacterial (Gram-positive) and lupanes. Examples of oleanane triterpenes with antibacterial activities are β-amyrin (**91**) and aceriphyllic acid A (**92**) (*Aceriphyllum rossii* (Oliv.) Engl.; Saxifragaceae; Core Eudicots)]. Antifungal oleananes are present in the resin of *Liquidambar formosana* Hance (Altingiaceae) [89,140,141,142,143,144,145,146,147,148,149,150,151,152,153,154,155]. In Malvids, gypsogenin (**93**) inhibited Gram-positive bacteria [155]. *Seco*-oleanane type triterpenes in the genus *Dysoxylum* Bl. are active against Gram-positive bacteria as seen with dysoxyhainic acid I (**94**) [156].

Oleanane triterpene saponins are active against yeasts and filamentous fungi [157,158,159,160,161,162]. The taraxasterane triterpene taraxerone (**95**) in *Schleichera oleosa* (Lour.) Oken (Sapindaceae) is antibacterial [163,164,165,166]. Ursanes are antibacterial, antimycobacterial, and antifungal [167,168,169,170]. The friedelane-type triterpene friedelin (**96**), from a plant in the genus *Polyalthia* Bl., inhibited *E. coli* (ATCC 25922) and *Micrococcus tetragenus* (ATCC 13623) [171]. *Tripterygium wilfordii* Hook. f. (Celastraceae) yields pristimerin (**97**), active against Gram-positive bacteria and fungi [172,173,174]. From *T. wilfordii*, celastrol (**98**) is antifungal and bacteriostatic for Gram-positive bacteria as well as zeylasterone (**99**) in *Kokoona zeylanica* Thwaites (Celastraceae), and the latter is a fungistatic for *C. albicans* [175,176]. Lanostane triterpenes from *Oenothera biennis* L. (Onagraceae; Malvids) were active against Gram-negative bacteria [177]. Cycloartanes such as (20*R*)-3*β*-hydroxy-24,25,26,27-tetranor-5*α*-cycloartan-23,21-olide (**100**) (Meliaceae) inhibit Gram-positive bacteria including MRSA [178]. Tirucallanes are antibacterial and antimycobacterial [179].

Plants in the Cucurbitaceae family (Fabids) yield antibacterial and antimycobacterial cucurbitanes [167] and multifloranes (bryononic acid) (**101**) [180,181]. Plants in the Meliaceae and Rutaceae produce broad-spectrum antibacterial limonoids such as 7-cinnamoyltoosendanin (**102**) and swietenolide (**103**), the latter active bacteria resistant to multiple antibiotics [182,183,184,185,186,187,188]. Limonoids are antifungal such as mulavanin D (**104**) and 2-hydroxyfissinolide (**105**) [163,189,190]. In the Simaroubaceae, quassinoids with antibacterial activity have been identified such as (6*R*)-methoxyjavanicin B (**106**) [191,192].

Steroids and steroidal saponins of the cholestane, ergostane (ergosterol-5,8-endoperoxide (**107**), stigmastanes [ campestane, furostane (dioscin) (**109**), pregnane, and cardenolide type are antibacterial, antimycobacterial, and antifungal] [193,194,195,196,197,198,199,200,201,202,203,204,205,206,207,208,209,210].

### 5.2. Miscellaneous

These are antibacterial malabaricans, oroceranes (*Lansium domesticum* Corrêa; Meliaceae), and ceanothanes such as zizimauritic acid A (**110**) [211,212,213,214].

## 6. The Distribution of Antibacterial and Antifungal Terpenes

Regarding the distribution of antibacterial and antifungal terpenes among Asian medicinal Angiosperms, it can be seen in Table 1 that all clades, except the Rosids, yield antibacterial and/or antifungal terpenes. Clades in the Core Eudicots tend to synthesise specific classes of antibacterial and/or antifungal terpenes such as dihydroagarofurans, jatrophanes, cassanes, and cucurbitanes (Fabids), or santalanes, quassinoids, and limonoids (Malvids). The Malvids are home to the broadest array of antibacterial and antifungal sesquiterpenes and triterpenes. In the Upper Angiosperms, Lamiids bring to being the broadest array of antibacterial and antifungal diterpenes. Antibacterial and antifungal terpenes with MIC ≤2 µg/mL are produced by plants in all three groups of Angiosperms.

## 7. Antibacterial and Antifungal Strength and Spectrum of Terpenes

Several guidelines are available to define the antibacterial strength of secondary metabolites from plants [6,7,8,9]. Here, a terpene is very strongly antibacterial (or antifungal) for MIC ≤ 2 µg/mL; strongly antibacterial (or antifungal) for a MIC > 2 µg/mL and ≤50 µg/mL; moderately antibacterial (or antifungal) for MIC > 50 and ≤100 µg/mL; weakly antibacterial (or antifungal) for a MIC > 100 and ≤500 µg/mL; very weakly antibacterial (or antifungal) for a MIC > 500 and ≤2500 µg/mL; inactive for a MIC > 2500 µg/mL and above.

For terpenes liquid at room temperature, we suggest very strong activity for a value below or equal to 2 µL/mL. According to Tampieri et al. (2005), strong activity is defined for natural products with MIC values ≤ 50 ppm [16]. Here, a terpene is defined as having moderate activity for MIC > 50 and ≤ 100 ppm; weak activity for MIC > 100 and ≤1500 ppm and inactivity for MIC > 1500 ppm.

Accordingly, out of about 300 antibacterial and/or antifungal terpenes identified between 1968 and 2022, 18 (four monoterpenes, five sesquiterpenes, four diterpenes, and five triterpenes) exhibited very strong activities (Table 2). Most of these were active against Gram-positive bacteria, followed by Gram-negative bacteria, mycobacteria, filamentous fungi, and yeasts.

## 8. Influence of Molecular Mass

The molecular mass of natural products influences their ability to fit in the catalytic pockets of enzymes, cytoplasmic membrane, and to cross the outer membrane via porins. Here, a low molecular mass was defined as below 200 g/mol, medium molecular mass from 200 to 400 g/mol, and high molecular mass above 400 g/mol. Following this classification, terpenes with MIC ≤ 2 µg/mL have a molecular mass ranging mainly from about 150 to 550 g/mol (Table 2).

Terpenes with low molecular mass are active against both Gram-positive and Gram-negative bacteria. It can be argued that being volatile, monoterpenes evaporate from paper discs or agar wells or even liquid broths explaining low activities recorded by most authors, except for Orhan et al., using emulsions [15,186]. Furthermore, the determination of MIC in the liquid broth of non-polar terpenes is almost impossible because they do not dissolve in an aqueous broth and we suggest using paper discs or dissolving the terpenes in melted solid agar for test in Petri dishes for this purpose as well as for measuring synergistic activities. Dimethyl sulfoxide has been recommended to facilitate the dissolution of non-polar natural products in liquid broth, but it has antibacterial and cytotoxic effects and does not dissolve most non-polar extracts and terpenes (personal communication).

Medium molecular mass is beneficial for activity against yeasts whereas filamentous fungi are sensitive to terpenes with low, medium, and high molecular masses.

Six out of the 18 terpenes with MIC ≤ 2 µg/mL had a high molecular mass and terpenes with a high molecular mass were only active against Gram-positive bacteria, probably because of their inability to cross porin channels. It can be observed that terpenes with strong activity against mycobacteria have medium to high molecular masses.

## 9. Influence of Solubility and Polar Surface Area

Water-soluble and amphiphilic terpenes cross porin channels [4]. Here, we define (at pH 7.4) terpenes with LogD below 1 as hydrophilic, LogD between 1 and 5 amphiphilic, andLogD above 5 as liposoluble.

Accordingly, it can be observed in Table 2 that there are no hydrophilic terpenes with MIC < 2 µg/mL. Amphiphilic terpenes are active against both Gram-positive and Gram-negative bacteria. Lipophilic terpenes are active against Gram-positive bacteria, specifically against mycobacteria, as they might dissolve into mycolic acid. The solubility of terpenes does not influence the activity against filamentous fungi whereas yeasts are specifically sensitive to mid-polar terpenes. The polar surface area of terpene with very strong activity is around 20 Å²_._

## 10. Structure Activity and Mechanism of Action

Regarding the structure–activity relationship and mode of action of terpenes, a general observation is that aromaticity, planarity, and substitutions with hydroxyl, ketone, aldehyde, or carboxylic acid groups increase the antibacterial and antifungal activities of terpenes. The presence of peroxide and/or epoxide groups is beneficial for antibacterial and antifungal properties as seen is amblyone (**89**), 1,8-cineole (**27**), artemisinin (**60**), 17-hydroxyjolkinolide B (**68**), or ergosterol-5,8-endoperoxide (**107**). Lipophilic terpenes are often antimycobacterial [96].

Linear monoterpenes inhibit the growth of both Gram-positive and Gram-negative bacteria, suggesting the targeting of the cytoplasmic and/or outer membrane. They are ineffective against Mycobacteria. Cyclic monoterpenes are broad-spectrum antibacterial and antifungal, but not antimycobacterial. The antibacterial mode of action of monoterpenes invokes the destabilisation of the cytoplasmic membrane such as in α-terpineol (**24**), terpinen-4-ol (**25**), δ-terpineol (**26**), and 1,8-cineole (**27**). Specific mechanisms arise with aromaticity, as seen with thymoquinone (**36**), which inhibits *E. coli* ATP synthase and in *C. albicans* induces the generation of reactive oxygen species [32,33,37,43,215,216,217]. The antifungal mode of action of monoterpenes includes cytoplasm coagulation, hyphal lysis, cell membrane insults, and the leakage of cellular cytoplasmic components [34]. The fact that the reduction and oxidation or isomerization of monoterpene does not much influence their strength against a broad-spectrum of bacteria and fungi spectrum points to mainly non-specific mechanisms, and most probably, accumulation in and the destabilisation of cytoplasmic membranes.

Sesquiterpenes are mainly broad-spectrum antibacterial, antimycobacterial, and antifungal via a mix of non-specific and specific mechanisms. Non-substituted sesquiterpenes like α-humulene (**61**) non-specifically target the membrane of Gram-positive bacteria and increase the permeability and intracellular content leakage [217]. The cytoplasmic membrane is also one of the non-specific fungal targets of amphiphilic sesquiterpenes, as seen with polygodial (**64**) with *S. cerevisiae* [91] and cadinanes [73]. For linear sesquiterpenes, the oxidation of hydroxyl groups into aldehyde is detrimental for activity against filamentous fungi. The introduction of a lactone moiety in sesquiterpenes boosts their activity against filamentous fungi, as seen with costunolide (**45**), cynaropicrin (**46**), deacetylxanthumine (**47**), and isoalantolactone (**48**) [47]. α-Methylene lactone moieties open to form Michael-type amine adducts with bacterial and fungal amino acids and ribonucleic acids. Furanone moieties in the presence of metal ions generate reactive oxygen species, forming strand breaks and the formation of 8-hydroxy-2′-deoxyguanosine in microbial DNA. Planarity and aromaticity translate into strong antibacterial (Gram-positive) properties, as seen with mansonone F (**56**) [71] and gossypol (**52**), the latter targeting DNA polymerase [218,219,220,221]. Epoxide groups are favourable for activity against Gram-negative bacteria, as seen with artemisinin (**60**), via copper-mediated DNA damage [79].

Diterpenes inhibit the growth of bacteria, mycobacteria, and fungi. Carboxylic, aldehyde, and epoxide groups as well as furanone moieties are favourable for activity as in the case of 17-hydroxyjolkinolide B (**68**) and 16*α*-hydroxy-cleroda-3,13 (14)Z-diene-15,16-olide (**84**). In diterpenes, the presence of a furanone moieties favours the generation of reactive oxygen species targeting the DNA in bacteria [110]. The presence of epoxide and aldehyde groups favour membrane damage in the bacteria and yeasts. (*E*)-8β, 17-Epoxylabd-12-ene-15,16-dial (**77**) is bactericidal via disintegration of the cytoplasmic membrane of *S. aureus* (ATCC 6538) and *Y. enterolytica* (MTCC 859) with the MIC/MBC values of 3.3/6.7 and 3.3/3.3 µg/mL, respectively [109].

Triterpenes are active against Gram-positive and Gram-negative bacteria, mycobacteria, yeasts, and filamentous fungi. The mechanism of action of triterpenes involves both non-specific and specific mechanisms. Lipophilic or amphiphilic triterpenes tend to damage the membrane with subsequent leakage of intracellular K^+^, as seen with geranylgeraniol (**65**) and (*E*)-phytol (**66**) [95,222]. Triterpenes with benzoquinone moieties, the ketone moiety in ring A conjugated with double bonds and substitution with carboxylic acid groups are strongly active [145] and tend to target bacterial and fungal DNA and/or topoisomerases, as seen with celastrol (**98**) and zeylasterone (**99**) [175,176]. Zeylasterone (**99**) induces cell membrane alterations in *B. subtilis* [176]. Limonoids inhibit DNA polymerase [223]. An increase in the lipophilicity and presence of endoperoxide or epoxide groups are beneficial for antimycobacterial and anti-Gram-negative activities, as seen with epoxy dammaranes [141]. The catabolism of cholesterol in *M. tuberculosis* requires enzymes [197] targeted by triterpenes and steroids. Triterpene saponins tend to target Gram-positive bacterial surface sortases [198], and like dioscin (**109**), lethal for *C albicans* via the formation of complexes with ergosterol in the cell membrane of fungi leading to the formation of pores, the loss of membrane integrity, and the leakage of cytoplasmic content [198,199,205,206,224,225].

## 11. Antibiotic and Antifungal Potentiating Effects

Terpenes potentiate antibiotics or antifungal agents in vitro and via non-specific and/or specific mechanisms:

*Non-specific mechanisms:* This type of synergy includes, for instance, the destabilisation of cytoplasmic membranes in Gram-positive bacteria by lipophilic and amphiphilic terpenes, destabilising the cytoplasmic membrane, as seen with linear terpenes [201], linalool (**10**) [20,21], 3,4-*seco*-mansumbinoic acid (**90**), cucurbitacins [180], cedrelanol (**57**) [72], and myrcene (**10**) [12,201]. Cucurbitacin B, for instance, decreases the resistance of *S. aureus* towards tetracycline and oxacillin [180]. Saponins are antibiotic potentiators for both Gram-positive and negative bacteria [198,199]. Pristimerin (**97**) and lupanes target DNA machinery and alters the membrane permeability of *S. aureus* [172,173,174]. Steroidal saponins such as dioscin have both non-specific and specific mechanism [205,206,226]. Limonoids target DNA machinery [223]. In fungi, an example of a non-specific potentiator is polygodial (**64**) [91]. Isoalantolactone (**48**) is an example of antibiotic potentiator acting both non-specifically on cytoplasmic membrane and specifically on MCR-1 to potentiate the effects of Polymyxin towards *E. coli* [227]. For mycobacteria, an example of a non-specific rifampicin-potentiator is artemisinin (**60**), which generates reactive oxygen species (due to the epoxide moiety) [228].

*Specific mechanisms:* Antibacterial potentiators such as clerodanes [229], carnosic acid (**70**), and oleananes [230] inhibit bacterial and fungal efflux pumps. Tiglianes inhibit P-glycoprotein in HepG2/ADR cells, and as such, might be able to inhibit bacterial and/or fungal efflux pumps [231]. Clerodanes inhibit NorA efflux pumps in *S. aureus* [232]. Neuroactive terpenes tend to inhibit bacterial efflux pumps. An example of neuroactive natural products inhibiting bacterial NorA is the monoterpene indole alkaloid reserpine from *Rauvolfia serpentina* (L.) Benth. ex Kurz (Apocynaceae; Lamiids). Additionally, reserpine is a calcium channel antagonist as is the synthetic calcium channel antagonist verapamil [233,234,235,236]. The reason why the calcium channel antagonists inhibit the bacterial efflux pump is, at least in part, because of the correlations between the bacterial efflux pumps and bacterial calcium transport [237]. Specific potentiators interfere with the cytoplasmic membrane polarisation of bacteria or fungi, resulting in efflux pump inhibition, as seen with cardenolides [238] and sesquiterpene lactones [239]. Another interesting feature of terpenes, and especially diterpenes, is their ability to remove genes of resistance from the plasmids of Gram-negative bacteria [122].

## 12. The Safety Issues of Terpenes with Respect on Human Health

Terpenes are phytoalexins/phytoanticipins produced by plants to poison/repel microbes, other plants, and animals [240]. For instance, mansonone E is antifeedant and phytotoxic [68]. In humans, terpenes can induce allergies, irritations as well as renal, pulmonary, hepatic, neurological, or cardiovascular damage [241,242,243]. Cardenolides are cardiotoxic, and euphorbiaceous phorbol esters are tumorigenic. At the cellular level, toxic terpenes disrupt cytoplasmic membranes, generate reactive oxygen species, and impair mitochondrial function [244]. Planar terpenes targeting bacterial DNA are often cytotoxic [66] as well as jatrophanes, daphnanes [130], gypsogenin [155], quassinoids [191], and steroidal saponins [207]. Therefore, selectivity indices using mammalian cells in vitro or lethal doses 50% (LD_50_) in studies using rodent are advised. The use of brine shrimps (*Artemia salina*) to determine the toxicity of antimicrobial terpenes is very simple and inexpensive [143].

## 13. Concluding Remarks

Weinstein and Albersheim (1983) argue that antibacterial natural products from plants act via non-specific mechanisms preventing the development of resistance [245]. The medicinal Angiosperms of Asia and the Pacific generate an enormous diversity of antibacterial and antifungal terpenes acting via specific and/or non-specific mechanisms representing a vast source of potential antimicrobial leads. However, terpenes are often difficult to isolate and identify, tend not to have good oral bioavailability, and are often toxic. For these reasons, identifying antibacterial or antifungal terpenes of clinical systemic usefulness is like trying to find a few needles in a large haystack, but the search is worthwhile.

For the last decades, a huge research effort has been provided in an attempt to find antimicrobials from the medicinal plants used for the treatment of infectious diseases in Asia and the Pacific resulting in the identification of about 300 terpenes. Among these, carvacrol, celastrol, cuminol, dysoxyhainic acid I, *ent*-1β,14β-diacetoxy-7α-hydroxykaur-16-en-15-one, ergosterol-5,8-endoperoxide, geranylgeraniol, gossypol, 16*α*-hydroxy-cleroda-3,13 (14)Z-diene-15,16-olide, 7-hydroxycadalene, 17-hydroxyjolkinolide B, (20*R*)-3*β*-hydroxy-24,25,26,27-tetranor-5*α* cycloartan-23,21-olide, mansonone F, (+)-6,6′-methoxygossypol, polygodial, pristimerin, terpinen-4-ol, and α-terpineol are original chemical frameworks from which there is the potential for the development of lead antibacterial or antifungal drugs.

## Figures and Tables

**Table 1:** Distribution of antibacterial and or antifungal terpenes in medicinal Angiosperms in Asia and the Pacific.

Groups of Angiosperms	Clades	Type of Terpenes	References
Basal Angiosperms	Protomagnoliids	Monoterpenes	[1,2,3,4,5,6,7,8,9,10,11,12,13,14,15,16,17,18,19,20,21,22,23,24,25,26,27,28,29,30,31,32,33,34,35,36,37,38,39,40,41,42,43]
		Sesquiterpenes (Lindenanes)	[44,45,46,47,48,49,50,51,52,53,54,55,56,57,58,59,60,61,62,63,64,65,66,67,68,69,70,71,72,73,74,75,76,77,78,79,80,81,82,83,84,85,86,87,88,89,90,91,92,93,94]
	Magnoliids	Monoterpenes	[1,2,3,4,5,6,7,8,9,10,11,12,13,14,15,16,17,18,19,20,21,22,23,24,25,26,27,28,29,30,31,32,33,34,35,36,37,38,39,40,41,42,43]
		Sesquiterpenes (Bisabolanes, Eudesmanes, Guaianes, Germacranes)	[44,45,46,47,48,49,50,51,52,53,54,55,56,57,58,59,60,61,62,63,64,65,66,67,68,69,70,71,72,73,74,75,76,77,78,79,80,81,82,83,84,85,86,87,88,89,90,91,92,93,94]
		Diterpenes (Clerodanes, Kauranes, Trachylobanes)	[95,96,97,98,99,100,101,102,103,104,105,106,107,108,109,110,111,112,113,114,115,116,117,118,119,120,121,122,123,124,125,126,127,128,129,130,131,132,133,134,135,136,137,138,139]
	Monocots	Monoterpenes	[1,2,3,4,5,6,7,8,9,10,11,12,13,14,15,16,17,18,19,20,21,22,23,24,25,26,27,28,29,30,31,32,33,34,35,36,37,38,39,40,41,42,43]
		Sesquiterpenes (Guaianes, Germacranes, Humulanes)	[44,45,46,47,48,49,50,51,52,53,54,55,56,57,58,59,60,61,62,63,64,65,66,67,68,69,70,71,72,73,74,75,76,77,78,79,80,81,82,83,84,85,86,87,88,89,90,91,92,93,94]
		Diterpenes (Clerodanes, Kauranes, Rosanes)	[95,96,97,98,99,100,101,102,103,104,105,106,107,108,109,110,111,112,113,114,115,116,117,118,119,120,121,122,123,124,125,126,127,128,129,130,131,132,133,134,135,136,137,138,139]
		Triterpenes (Dammaranes, Stigmastanes, Spirostanes, Tirucallanes)	
Core Angiosperms	Eudicots	Monoterpenes	[1,2,3,4,5,6,7,8,9,10,11,12,13,14,15,16,17,18,19,20,21,22,23,24,25,26,27,28,29,30,31,32,33,34,35,36,37,38,39,40,41,42,43]
		Diterpenes (Diterpene alkaloids)	[95,96,97,98,99,100,101,102,103,104,105,106,107,108,109,110,111,112,113,114,115,116,117,118,119,120,121,122,123,124,125,126,127,128,129,130,131,132,133,134,135,136,137,138,139]
	Core Eudicots	Monoterpenes	
		Triterpenes (Lupanes, Oleananes)	
	Fabids	Monoterpenes	[1,2,3,4,5,6,7,8,9,10,11,12,13,14,15,16,17,18,19,20,21,22,23,24,25,26,27,28,29,30,31,32,33,34,35,36,37,38,39,40,41,42,43]
		Sesquiterpenes (Bisabolanes, Dihydroagarofurans)	[44,45,46,47,48,49,50,51,52,53,54,55,56,57,58,59,60,61,62,63,64,65,66,67,68,69,70,71,72,73,74,75,76,77,78,79,80,81,82,83,84,85,86,87,88,89,90,91,92,93,94]
		Diterpenes (Cassanes, Clerodanes, Jatrophanes, Kauranes, Pimaranes)	[95,96,97,98,99,100,101,102,103,104,105,106,107,108,109,110,111,112,113,114,115,116,117,118,119,120,121,122,123,124,125,126,127,128,129,130,131,132,133,134,135,136,137,138,139]
		Triterpenes (Cucurbitanes, Friedelanes, Lupanes, Oleananes, Stigmastanes, Ursanes)	
	Malvids	Monoterpenes	[1,2,3,4,5,6,7,8,9,10,11,12,13,14,15,16,17,18,19,20,21,22,23,24,25,26,27,28,29,30,31,32,33,34,35,36,37,38,39,40,41,42,43]
		Sesquiterpenes (Cadinanes, Drimanes, Eudesmanes, Germacranes, Guaianes, Humulanes, Santalanes)	[44,45,46,47,48,49,50,51,52,53,54,55,56,57,58,59,60,61,62,63,64,65,66,67,68,69,70,71,72,73,74,75,76,77,78,79,80,81,82,83,84,85,86,87,88,89,90,91,92,93,94]
		Diterpenes (Pimaranes)	[95,96,97,98,99,100,101,102,103,104,105,106,107,108,109,110,111,112,113,114,115,116,117,118,119,120,121,122,123,124,125,126,127,128,129,130,131,132,133,134,135,136,137,138,139]
		Triterpenes (Cholestanes, Dammaranes, Ergostanes, Lanostanes, Limonoids, Lupanes, Oleananes, Quassinoids, Stigmastanes, Taraxasteranes, Ursanes)	
Upper Angiosperms	Asterids	Diterpenes (Cadinanes, Clerodanes)	[95,96,97,98,99,100,101,102,103,104,105,106,107,108,109,110,111,112,113,114,115,116,117,118,119,120,121,122,123,124,125,126,127,128,129,130,131,132,133,134,135,136,137,138,139]
		Triterpenes (Oleananes)	
	Lamiids	Monoterpenes	[1,2,3,4,5,6,7,8,9,10,11,12,13,14,15,16,17,18,19,20,21,22,23,24,25,26,27,28,29,30,31,32,33,34,35,36,37,38,39,40,41,42,43]
		Sesquiterpenes (Eudesmanes, Guaianes, Humulanes)	[44,45,46,47,48,49,50,51,52,53,54,55,56,57,58,59,60,61,62,63,64,65,66,67,68,69,70,71,72,73,74,75,76,77,78,79,80,81,82,83,84,85,86,87,88,89,90,91,92,93,94]
		Diterpenes (Cembranes, Clerodanes, Kauranes, Labdanes, Scopaludanes)	[95,96,97,98,99,100,101,102,103,104,105,106,107,108,109,110,111,112,113,114,115,116,117,118,119,120,121,122,123,124,125,126,127,128,129,130,131,132,133,134,135,136,137,138,139]
		Triterpenes (Cardenolides, Ergostanes, Oleananes, Spirostanes, Ursanes)	
	Campanuliids	Monoterpenes	[1,2,3,4,5,6,7,8,9,10,11,12,13,14,15,16,17,18,19,20,21,22,23,24,25,26,27,28,29,30,31,32,33,34,35,36,37,38,39,40,41,42,43]
		Sesquiterpenes (Bisabolanes, Eudesmanes, Germacranes, Guaianes)	[44,45,46,47,48,49,50,51,52,53,54,55,56,57,58,59,60,61,62,63,64,65,66,67,68,69,70,71,72,73,74,75,76,77,78,79,80,81,82,83,84,85,86,87,88,89,90,91,92,93,94]
		Diterpenes (Diterpene alkaloids, Kauranes)	[95,96,97,98,99,100,101,102,103,104,105,106,107,108,109,110,111,112,113,114,115,116,117,118,119,120,121,122,123,124,125,126,127,128,129,130,131,132,133,134,135,136,137,138,139]

**Table 2:** Terpenes with very strong antibacterial and/or antifungal activities (MIC ≤2 µg/mL).

Type of Terpenes	Name of Terpenes	MM (g/mol)	LogD	PSA (Å²)	Gram-Positive	Gram-Negative	Mycobacteria	Filamentous Fungi	Yeasts	References
Monoterpenes	α-Terpineol (**24**)	154.2	3	20	*S. aureus* (*)	*E. coli* (*)		*G. citri-aurantii* (*)		[20,32]
	Terpinen-4-ol (**25**)	154.2	3		*S. aureus* (*)	*E. coli* (*) *S. enteritidis* (*)				[33,36]
	Cuminol (**32**)	150.2	2.3	20	*B. cereus*					[15]
	Carvacrol (**37**)	150.2	3	20	*B. subtilis*	*P. aeruginosa*				[13,16]
Sesquiterpenes	Gossypol (**52**)	518.5	5.1	156	*B. cereus* *S. aureus* *S. epidermidis*					[62,63,64,65,66,67,68,69,70]
	(+)-6,6′-Methoxygossypol (**53**)	546.7	n.a	n.a	*E. faecalis*					[62,63,64,65,66,67,68,69,70]
	7-Hydroxycadalene (**54**)	214.3	4.7	20	*B. cereus*					[62,63,64,65,66,67,68,69,70]
	Mansonone F (**56**)	240.2	2.5	43	MRSA					[71]
	Polygodial (**64**)	234.3	3.8	34				*S. libertiana*	*S. cerevisae* *H. anomala* *C. utilis*	[90,92]
Diterpenes	Geranylgeraniol (**65**)	290.4	7.4	20	*S. aureus*					[95]
	17-Hydroxyjolkinolide B (**68**)	346.4	2.6	72			*M. smegmatis*			[99]
	*ent*-1β,14β-diacetoxy-7α-hydroxykaur-16-en-15-one (**81**)	n.a	n.a	n.a			*M. tuberculosis*			[119]
	16*α*-Hydroxy-cleroda-3,13 (14)Z-diene-15,16-olide (**84**)	n.a	n.a	n.a		*E. coli* *K. pneumoniae* *P. aeruginosa* *S. typhi*				[119]
Triterpenes	Dysoxyhainic acid I (**94**)	458.7	n.a	57.5	*B. subtilis*					[156]
	Pristimerin (**97**)	464.2	7.1	64	*B. subtilis* (°) *S. epidermidis* (°)					[172,173,174]
	Celastrol (**98**)	450.6	4	75	*B. cereus* (°) *B. megaterium* (°) *B. pumilus* (°) *B. subtilis* (°) *S. aureus* (°) *S. epidermidis* (°)			*H. capsulatum* (*)	*C. neoformans* (*)	[175]
	(20*R*)-3*β*-Hydroxy-24,25,26,27-tetranor-5*α* cycloartan-23,21-olide (**100**)	n.a	n.a	n.a	MRSA					[178]
	Ergosterol-5,8-endoperoxide (**107**)	412.6	6.9	29			*M. tuberculosis*			[194,195,196,197]

MM: molecular mass; PSA: polar surface area; (*): bactericidal or fungicidal; (°): bacteristatic or fungistati.

## Data Availability

Not applicable.

## Supplementary Materials

The following supporting information can be downloaded at: https://www.mdpi.com/article/10.3390/molecules28093873/s1, Table S1: Antibacterial and antifungal terpenes from the medicinal plants of Asia and the Pacific.
