# Supplementary material for: Antibacterial and Antifungal Terpenes from the Medicinal Angiosperms of Asia and the Pacific: Haystacks and Gold Needles
Source: Molecules. 2023 May 4;28(9):3873. doi: 10.3390/molecules28093873 (PMC10180233; doi:10.3390/molecules28093873)
Supplement: Supplementary file 1 [file molecules-28-03873-s001.zip › molecules-2130840-supplementary.pdf]

Antibacterial and Antifungal Terpenes from the Medicinal Angiosperms of Asia and the Pacific: Haystacks and Gold Needles

Table S1. Antibacterial and antifungal terpenes from the medicinal plants of Asia and the Pacific

TYPE OF TERPENES	NAME OF TERPENES	Micro-organism	MIC (μg/mL)	MBC/MFC	References
**MONOTERPENES**	Nerol (**2**)	*C. albicans*	200*		[16]
	Geranial (**5**)	*C. albicans*	100*		[16]
	Citronellol (**6**)	*Trichophyton sp.*	34		[14]
	Linalool (**9**)	*C. albicans*	500*		[16]
	α-Terpineol (**24**)	*E. coli* (ATCC 25922)	0.7	0.7	[32]
		S. enteritidis	3.1	3.1	[32]
		S. aureus (ATCC 25923)	1.5	3.1	[32]
		G. citri-aurantii	2°	4°	[34]
	Terpinene-4-ol (**25**)	*E. coli* (ATCC 25922)	1.5°	1.5°	[32]
		S. enteritidis	3.1°	3.1°	[32]
		S. aureus (ATCC 25923)	1.5°	1.5°	[32]
	1,8-Cineole (**27**)	*E. coli* (ATCC 25922)	3.1°	3.1°	[37]
		S. enteritidis	6.2°	6.2°	[37]
		S. aureus (ATCC 25923)	6.2°	6.2°	[37]
		furfur (ATCC 44344)	62.5		[37]
	γ-Terpinene (**28**)	*S. enteritidis*	3.1°		[32]
	α-Terpinene	*C. albicans*	100*		[16]
	p-Cymene (**31**)	*C. albicans*	100*		[16]
	Cuminol (**32**)	*B. subtilis* (ATCC 6633)	2		[15]
	Thymol (**33**)	*C. albicans* (ATCC 10231)	8		[15]
		8 *C. parapsilosis* (ATCC 22019)	16		[15]
	Thymoquinone (**36**)	*B. cereus*	8	8	[40]
		S. aureus	8	16	[40]
		C. dublinensis	25		[43]

	Carvacrol (**37**)	*P. aeruginosa* (ATCC 10145)	2		[15]
**SESQUITERPENES**	Farnesol (**38**)	*Trichophyton sp.*	3.5		[14]
		C. albicans	35		[14]
	Farnesal (**39**)	*S. aureus*	40		[14]
		MRSA	40		[14]
	Germacrone (**40**)	*P. aeruginosa*	15.6	31.2	[46]
	Costunolide (**45**)	*T. simii*	31.2		[47]
	Deacetylxanthumine (**47**)	*P. dreschleri*	12.5		[54]
	Microjaponin (**49**)	*M. tuberculosis*	12.5		[60,61]
	8-Acetoxymutangin (**50**)	*M. tuberculosis*	35		[60]
	Mochinine H (**51**)	*M. orizae*	19.7		[62,64]
	Gossypol (**52**)	*S. aureus* (TISTR517)	1.1		[67]
		B. cereus	1.1		[68,70]
		E. faecium (VRE)	2.5	10	[68,70]
		T. mentagrophytes	12.5		[68,70]
		M. canis	25		[68,70]
		T. rubrum	50		[68,70]
		M. gypseum	100		[68,70]
	(+)-6,6′-Methoxygossypol (**53**)	*B. cereus*	2.3		[68,70]
	7-Hydroxycadalene (**54**)	*B. cereus*	0.5		[68,70]
	Mansonone E (**55**)	*C. gloeosporioides*	31.2	31.2	[71]
		P. parasitica	31.2	125	[71]
	Mansonone F (**56**)	MRSA	2		[71]
		B. subtilis (ATCC 6633)	2		[71]
		S. aureus (ATCC 25223)	1		[71]
		S. epidermidis (ATCC 12228)	0.5		[71]
		K. pneumoniae (ATCC 10031)	8		[71]
	Cedrelanol (**57**)	*S. aureus*	4		[72]
		T. mentagrophytes	2.3		[72]
	α-Humulene (**61**)	*S. lutea* (IFO 3232)	3.9		[79]
		B. subtilis (IFO 3026)	3.9		[79]
		X. campestris	7.8		[79]
		M. tuberculosis (H37Ra)	6.2		[80]
	α-Santalol (**62**)	*T. rubrum*	12.5		[87]
	β-Santalol (**63**)	*T. rubrum*	25		[87]
	Polygodial (**64**)	*S. cerevisae* (IFO 0203)	0.7		[91,92]

		H. anomala (IFO 0136)	1.5		[91,92]
		C. utilis (ATCC 42402)	1.5		[91,92]
		S. libertiana	1.5		[91,92]
**DITERPENES**	Geranylgeraniol (**65**)	*S. aureus* (FDA209P)	< 1.2		[95]
	(*E*)-Phytol (**66**)	*M. tuberculosis* (H37Rv)	32		[96]
	Toonaciliatin M (**67**)	*T. rubrum*	12.5		[98]
	17-Hydroxyjolkinolide B (**68**)	*M. smegmatis*	1.5		[99]
	6*β*-Cinnamoyl-7β-hydroxyvouacapen-5*α*-ol (**73**)	*Mycobacterium sp.*	6.2		[105]
	Niloticane (**74**)	*B. subtilis*	4		[106]
		S. aureus	8		[106]
		K. pneumoniae	16		[106]
		E. coli	3		[106]
	Neocaesalpin P (**75**)	*S. aureus*	16		[107]
		S. agalactiae	16		[107]
		P. aeruginosa	32		[107]
	(*E*)-8β, 17-Epoxylabd-12-ene-15,16-dial (**77**)	*S. aureus* (ATCC 6538)	3.3	6.7	[109]
		Y. enterolitica	3.3	3.3	[109]
	ent-Trachyloban-19-oic acid (**78**)	*M. smegmatis*	6.2		[113]
		S. mutans	8.9		[114,115]
		P. gingivalis	57.6		[114,115]
	ent-Kaur-16-en-19-oic acid (**79**)	*M. smegmatis*	6.2		[118]
	ent-18-acetoxy-7*α*-hydroxykaur-16-en-15-one (**80**)	*M. tuberculosis* (H37Ra)	3.1		[119]
	ent-1β,14β-Diacetoxy-7*α*-hydroxykaur-16-en-15-one (81)	*M. tuberculosis* (H37Ra)	1.5		[119]
	Bafoudiosbulbin C (**83**)	*M. smegmatis* (ATCC 700084)	8	16	[121]
		M. tuberculosis (ATCC 27294)	8	8	[121]
	16*α*-Hydroxy-cleroda-3,13 (14)Z-diene-15,16-olide (84)	*S. aureus*	6.2		[125]
		E. coli	0.7		[125]
		P. aeruginosa	0.7		[125]
		S. typhi	0.7		[125]
		K. pneumoniae	1.5		[125]
	16-Oxo-cleroda-3, 13(14) *E*-diene-15 oic acid (**85**)	*S. aureus*	12.5		[125]
	Euphoheliosnoid E (**86**)	*A. viscosus* (ATCC 27044)	3.9		[133]
	Dehydroabietylamine (**88**)	*S. aureus*	12.5		[139]
		P. aeruginosa	6.2		[139]
	3,4-*seco*-Mansumbinoic acid (**90**)	*S. aureus* (1199B)	4		[142]
**TRITERPENES**	Aceriphyllic acid A (**92**)	*S. aureus* (503)	4		[153]

		MRSA (CCARM 3167)	8		[153]
		S. aureus (CCARM 3505)	8		[153]
		B. subtilis (KCTC 1021)	8		[153]
		A. calcoaceticus	4		[153]
		M. luteus (KCTC 1056)	8		[153]
	Dysoxyhainic acid I (**94**)	*B. subtilis*	1.5		[156]
	Taraxerone (**95**)	*C. gloeosporioides*	5		[163-166]
		T. halophyllus (ATCC 13623)	5		[171]
	Pristimerin (**97**)	*B. subtilis*	1.2	5	[172]
		S. epidermidis	1.2	>40	[172]
		H. capsulatum (0G217B)	0.4	0.4	[173,174]
		C. neoformans	7.8	7.8	[173,174]
		C. krusei (ATCC 6258)	7.8	62.5	[173,174]
	Celastrol (**98**)	*B. subtilis*	0.1	2.5	[175]
		B. cereus	0.6	2.5	[175]
		B. pumilus	0.6	2.5	[175]
		B. megaterium	1.2	5	[175]
		S. aureus	1.2	5	[175]
		S. epidermidis	0.3	15	[175]
	Zeylasterone (**99**)	*C. albicans*	10	40	[176]
	(20*R*)-3*β*-Hydroxy-24,25,26,27-tetranor-5*α*-cycloartan-23,21-olide (**100**)	MRSA	1.5		[178]
	Bryononic acid (**101**)	*S. enteritica*	6		[181]
	Ergosterol-5,8-endoperoxide (**107**)	*Mycobacterium sp.*	1		[194]

*:p.p.m.; °: µL/mL
